# Caregiving for Patients With Frontotemporal Dementia in Latin America

**DOI:** 10.3389/fneur.2021.665694

**Published:** 2021-07-07

**Authors:** Stefanie Danielle Piña-Escudero, Gloria Annette Aguirre, Shireen Javandel, Erika Mariana Longoria-Ibarrola

**Affiliations:** ^1^The Global Brain Health Institute, University of California, San Francisco, San Francisco, CA, United States and Trinity College, Dublin, Ireland; ^2^Instituto Nacional de Ciencias Médicas y Nutrición Salvador Zubirán, Mexico City, Mexico; ^3^Department of Neurology, Memory and Aging Center, University of California, San Francisco, San Francisco, CA, United States; ^4^Instituto Nacional de Neurología y Neurocirugía José Velazco Suárez, Mexico City, Mexico

**Keywords:** caregivers, frontotemporal dementia, Latin America, caregiver burden, dementia

## Abstract

Latin America is a vast heterogeneous territory where chronic diseases such as mild cognitive impairment or dementia are becoming higher. Frontotemporal dementia (FTD) prevalence in this region is estimated to be around 12–18 cases per thousand persons. However, this prevalence is underestimated given the lack of awareness of FTD even among healthcare professionals. Family members are responsible for the care of patients with FTD at home. These caregivers deliver care despite being ill-equipped and living in the context of austerity policies and social inequities. They often face unsurmountable financial and social burdens that are specific to the region. The most important step to support caregivers in Latin America is to increase awareness of the disease at all levels. Healthcare diplomacy is fundamental to create joint efforts that push policies forward to protect caregivers of FTD patients.

## Introduction

In Latin America, dementia numbers are rapidly rising (1). The incidence of dementia diagnosis has been estimated between 9.10 and 13.8 per 1,000 people/year (2, 3). The global dementia prevalence in Latin American among older adults is 11% with Alzheimer's Disease being the most frequent type (4). In Latin American middle-income countries, the mortality risk is 1.56–5.69 times higher than in individuals without dementia (5). FTD is a term used to encompass the clinical syndromes that result from frontotemporal lobar degeneration pathology. These clinical syndromes include the behavioral variant of FTD (bvFTD), semantic and non-fluent variants of primary progressive aphasia (PPA), and the overlap syndromes with amyotrophic lateral sclerosis (FTD-ALS) or with other parkinsonian syndromes (i.e., corticobasal syndrome, progressive supranuclear palsy) (6). There is little information about the prevalence of FTD in Latin America, but it is estimated to be around 12 to 18 cases per thousand persons in community-dwelling settings (7), which is in line with estimates in other countries (10.84/100,000) (8, 9). In high-income countries, studies of FTD have increased considerably over the last decade and FTD is now considered the second leading cause of dementia for those under 65 years (10), thanks to the many advances in neuropathology, biochemistry, and genetics that clarified correlations between molecular profiles and clinical phenotypes (11).

In contrast, in Latin America, these resources are extremely limited and most healthcare professionals, even specialists, are unaware of FTD and lack the training to diagnose it (12, 13). The model of memory clinics that is commonly found in developed countries is extremely limited in Latin America and constrained mostly to big cities (14). In rural settings, economic and geographical barriers limit access to specialized healthcare even more (15). Scarce financial and social resources, limited knowledge about the disease, and competing health and social needs challenge caregivers around the world, however, in low-income countries, inequality has substantially affected capacity building for diagnosis and follow-up care in FTD. In Latin America, informal employment is the source of income of more than half of the households, resulting in limited access to medical services, disability insurance, and other benefits associated with formal employment (16, 17). The few public services that ensure continuity of care for patients such as in-home care, long-term care, and palliative care cover only a very small sector of the population (18). Additionally, few private resources are available and are accessible only for patients who have financial resources to cover the high costs (19). In high-income countries, the estimated annual cost for care for patients with FTD can be over $100,000 (twice the cost of care for a patient with Alzheimer's disease) (20). In Latin America, FTD care represents an insurmountable financial burden.

### Caregiving for FTD Patients in LAC

Since research in FTD is a niche science in Latin America with only a few published manuscripts, research on caregiving in FTD is practically non-existent in the region. In fact, we conducted a literature search of the published literature on PubMed, EMBASE, and Web of Science databases up to April 2021. The search strategy that retrieved the majority of the studies used the keywords “caregiver burden” AND “frontotemporal dementia.” From the 268 retrieved articles, just two of them were conducted in Latin America and just one covered the relationship between caregivers and patients with FTD. Due to the scarcity of published work around this topic, our effort was focused on contextualizing the challenges and lived experiences of caregivers of patients with FTD in Latin America, setting the stage for future avenues of research on this topic.

In Latin America, most of the caregivers for patients with FTD are family members. They compensate for the lack of formal governmental support and insufficient financial resources by taking care of patients with FTD at home. This responsibility can consume more than 8 h a day (21). Often influenced by cultural factors, caregiver roles are mainly fulfilled by females (22, 23). These women oftentimes have low education and live in multigenerational households where taking care of the patient is not their main role (21, 22). Female caregivers have to play multiple roles such as wives, daughters, mothers, or employees, and more often than is the case with men, women's caregiving roles interfere with other life activities, which may ultimately lead to “role-captivity” (caregiver feelings of being “trapped” in their role) (24). Caregivers frequently take care of patients at home, setting an example for their children about family obligation and intergenerational reciprocity (25). In the context of FTD, it is important to account that genetic variants of the disease such as mutations in C9ORF72, MAPT, GRN, TARDBP, etc. have been described to affect large families in Latin America and a caregiver may be in charge of the care of multiple sick members of a family (26).

### Challenges for Caregivers of Patients With bvFTD

The trajectory of perceived caregiver burden depends largely on the clinical phenotype of the patient and the practical daily issues that derive from each syndrome (27). bvFTD is characterized by behavioral disinhibition, apathy, loss of empathy, perseverative, stereotyped, or compulsive/ ritualistic behaviors, binge eating behavior, and dietary changes (28). Common behaviors include inappropriate sexual conduct, shoplifting, aggression, loss of manners, and tactlessness. The study of Lima Silva et al. showed that, compared to Alzheimer's disease, Brazilian participants with FTD presented more agitation/aggression (0.001), apathy (<0.001), disinhibition (<0.001), euphoria (0.021), and irritability (0.003) (29). All of these behavioral symptoms place a heavy toll on caregivers from the beginning of the patient's disease. Brazilian caregivers of participants with FTD doubled the distress scores of their counterparts caring for participants with Alzheimer's disease measured by the Caregiver Distress Scale part of the Neuropsychiatric Inventory obtaining 13.22 (±7.94) vs. 6.13 (±4.67) points respectively with a *p* < 0.001. The participant's symptoms that generated the statistically significant differences in caregivers' distress were apathy, disinhibition, irritability, and aggression (29). It can be particularly emotionally taxing when caregivers do not understand these behaviors to be symptoms of dementia because of cultural views or because it is difficult to obtain a diagnosis from a healthcare provider.

Since behavioral symptoms often present in patients before the age of 65 without other neurological and/or cognitive complaints (28), they are frequently not interpreted as pathological by physicians. The study conducted by Gleichgerrcht et al. showed that <30% of general practitioners in Latin America have heard of bvFTD during their medical training (12). Coping with a patient presenting misunderstood behavioral symptoms can lead to emotions of shame, irritation, guilt, exhaustion, and fear in the caregivers. These negative emotions can affect their interpersonal relationships with the patients and even lead to mistreatment (30, 31). In Latin America, there is also a strong culture of unconditional respect for the patriarch. Women and young family members caring for an older male may find it uncomfortable to redirect inappropriate behaviors, not wishing to be seen as lacking respect (32, 33). Further complicating matters, caregivers may be less likely to seek professional help if the presenting behavioral symptoms consist of inappropriate sexual comments, excessive drinking, and/or aggressivity, as these actions are often dismissed or even accepted within Latin American cultures. As a result of this sociocultural setting, caregivers themselves may see the behaviors as intentional, not recognizing them as symptoms of bvFTD (33), potentially delaying interventions of benefit to the patient. On the positive side, since pathological behaviors are within the accepted social norms, caregivers might experience less burden when caring for these patients. This could be a possible explanation for the unexpected findings of Lima-Silva et al. that reported that in a predominantly masculine cohort in Brazil, caring for participants with FTD with behavioral symptoms, even when more distressful, was not more burdensome than caring for patients with Alzheimer's disease measured by the Zarit Burden Inventory (*p* = 0.150) (29). If these same behaviors are present in women with FTD, particularly impulsivity related to sexual behaviors, caregivers may feel shame and isolate themselves and the patients from society to avoid public embarrassment (34, 35). The CUIDEME Study reported that in a predominantly female cohort with dementia in Chile, a higher number of neuropsychiatric symptoms in the Neuropsychiatric Inventory correlated with a higher caregiver burden (*p* < 0.001) (23). Chronic stress from constant aggression and social isolation increase burden and the caregiver's risk for physical and mental illness (36).

There can also be a substantial financial burden of caring for patients with bvFTD, even when families are not paying for formal care. Symptoms like apathy, impulsivity, inability to engage in complex activities can result in loss of employment early in the course of the disease. With a relatively young age of onset, many families of patients with bvFTD are still reliant on the patient's income and this loss can represent a considerable financial strain as the family will need to take care of the patient while establishing a new primary source of income. Furthermore, family members who become caregivers have less opportunity to advance their own careers or to support the educational or career advancement of their children (21). The COVID-19 pandemic has been especially challenging for the caregivers of patients with bvFTD as behavioral and cognitive symptoms make these patients less likely to follow the safety recommendations, putting themselves and their caregivers at a higher risk of contracting the virus.

Limited public healthcare resources and the high cost of private care can also increase the burden that caregivers of patients with bvFTD experience. Patients with bvFTD are usually referred for a psychiatric evaluation as behavioral symptoms progress (37). For many caregivers, getting the patient to specialized medical care represents a huge challenge. Mental health services in general hospitals are very limited. Outside of Argentina and Uruguay, Latin American countries have fewer than 10 psychiatrists per 100,000 citizens (38). Therefore, families who do not live in urban areas often need to travel long distances to receive this specialized care and they must cover those expenses out of pocket, which can present a high financial burden. Additionally, the proportion of psychiatrists in Latin America who answered affirmatively to whether they diagnose dementia went from (yes to no ratio) 6:1 to 1.49:1 when asked if they make the differential diagnosis of bvFTD (12). Therefore, since few psychiatric specialists are trained to identify bvFTD, diagnosis may be delayed, further referrals could be requested, or the patient could be misdiagnosed with a psychiatric disorder (31). Misdiagnosis or delay in correct diagnosis reduces the caregiver's ability to understand the patient's symptoms and seek any supportive resources that could exist locally, further increasing the burden of care (39). Fortunately, some countries including Mexico, Costa Rica, Colombia, and Chile, have begun incorporating psychiatrists in their memory clinics, which is slowly increasing awareness of bvFTD in the field.

In terms of services for caregivers that could mitigate the burden, only a handful of specialized support groups for patients with bvFTD can be found and exist mainly in big cities in Brazil,^1^ Argentina,^2^ and Colombia.^3^ Most of these groups are focused on creating printed materials and informative sessions to support caregivers. The content of these sessions is mostly focused on decreasing the burden of care by offering techniques to manage difficult behavioral symptoms of patients. Unfortunately, there are still places like Nicaragua where, due to the lack of appropriate FTD diagnosis, the creation of services and resources has been stymied. Frequently, family caregivers who have already lost income to provide care, face the additional expense of hiring outside caregivers to further assist (21). In Uruguay, the government subsidizes costs for in-home care assistance for people with disabilities. However, in this system, the priority is given to people under 30 years of age and over 80 in a situation of severe dependency, and people over 65 in a situation of mild and moderate dependency so, it is not ideal for bvFTD where symptom onset occurs between those ages (40). Frequently, family caregivers who have already lost income to provide care, face the additional expense of hiring outside caregivers to further assist (21).

For many caregivers of patients with bvFTD, as the disease progresses, the burden becomes greater, and the social network becomes more limited. In this situation, institutionalization may be considered as the last resource (25). There are a limited number of long-term care institutions in Latin America. Governmental support is extremely limited and private markets are not regulated, allowing private facilities to charge more to care for people with behavioral symptoms. Most families willing to access these services would need to pay high out-of-pocket costs that are not possible for most middle- and low-income families (41, 42). Caregivers must also contend with the reluctance of patients with bvFTD to be institutionalized and a sense of guilt in the context of cultures that view institutionalization as a form of disrespect or betrayal (43). Since most of these institutions are designed to care for older patients, individuals with bvFTD may lack a sense of belonging. Frequently, patients with bvFTD end up in psychiatric institutions. Brazil and Chile, the countries with the highest number of psychiatric beds in public long-term care facilities, provide only 0.3 beds per 100,000 population and they are not exclusive or specifically equipped to care for patients with bvFTD (38).

### Challenges for Caregivers of Patients With Other Forms of FTD

In contrast to bvFTD where caregiver burden is higher earlier in the course of the disease, in PPA and ALS, caregiver burden tends to increase over time (27). Semantic (sv) and non-fluent variants of PPA are typically characterized by language impairments when they affect the left side of the brain (44). Although not a Latin American example, a study conducted by Koyama et al. in Japan, compared the Zarit Burden Interview score from caregivers of participants mostly in mild stages of right svPPA, left svPPA, and bvFTD. Caregivers of participants with bvFTD reported the highest Zarit Burden Interview scores (0.002). No significant differences in ZBI scores were found in the right vs. left svPPA (*p* = 0.166). However, the effect size was large (*d* = 0.89) (45). These results aside, behavioral symptoms do emerge with disease progression and they can be somewhat unexpected for the caregivers. When behavioral symptoms emerge, caregivers might experience a greater sense of burden since they will no longer be caring for a family member with only a language difficulty (27). Individuals with right-sided predominant semantic variant PPA exhibit prosopagnosia and early behavioral changes similar to those seen in bvFTD, such as social awkwardness, job loss, loss of insight, and difficulty with personal identification (46). Their caregivers will experience similar challenges to those experienced by caregivers of patients with bvFTD. In the study conducted by Hsieh et al. in an Australian population, the authors showed an increase in behavioral symptoms and the Zarit Burden Interview score (*p* < 0.001) over a 3-year follow-up period in participants with svPPA compared to their caregivers of bvFTD participants counterparts whose Zarit Burden Interview score remained high throughout the follow-up (27).

As with bvFTD, caregivers of patients with PPA face difficulties in getting an early and accurate diagnosis. Aphasia might be incorrectly attributed to stroke or trauma, especially in rural settings where brain imaging is not available (12, 47). Importantly, the svPPA diagnostic criteria were developed for English speakers, and challenges with applying these criteria in patients who speak Portuguese, Spanish or indigenous languages can delay diagnosis (48, 49).

In Latin America, the family unit is the central part of society and an essential element of well-being and it is considered necessary to provide optimal care to the members who need it. Tight family bonds are built through communication between its members. Breakdowns in communication resulting from language deficits could lead to a loss of the sense of family and result in social isolation among caregivers and patients (50, 51). As the patient loses the ability to communicate, family roles need to change, especially in those circumstances in which the patient lost his or her job as a consequence of aphasia (51). This is especially important for very traditional Latin American families with very pre-determined gender roles in which spouses are not equipped with the skills to fulfill their partner's role. Fulfilling opposite gender roles might lead to frustration, stress, anxiety, and embarrassment that importantly increase caregiver burden (52). For Latin American indigenous families, language plays a critical role in sustaining the ancient culture. Oral tradition represents an important part of the inheritance from one generation to the next. Caregivers who are children of people with PPA are unable to receive that knowledge and can experience additional frustration and guilt for not being able to carry and transmit the heritage of their family line (53, 54).

Speech therapy can ameliorate the burden of the disease for patients while also providing indirect relief to caregivers. Brazil has made an important effort to increase the number of phonoaudiologists and language therapists and to raise awareness of PPA among this group. While speech rehabilitation services can be effective at addressing symptoms, they are limited in Latin America (55, 56). Other barriers to access include financial constraints, caregiver availability and transportation limitations. As improvements will be short-term in the context of a disease that will inevitably result in language deterioration, the costs for caregivers in participating in these programs may outweigh the benefits (57, 58). Notably, some online programs have emerged during the COVID-19 pandemic that may provide solutions for geographical issues (see text footnote 2, 3).

Patients with FTD-ALS experience the shortest mean and median survival of the FTD subtypes (59). Each patient displays a unique set of symptoms that come with the motor manifestations such as changes in behavior, personality, and language skills. As pointed out by Hsieh et al., caregivers of participants with FTD-ALS can experience a steeper caregiver burden increase compared to patients with svPPA and bvFTD over a 3-year follow up. High Zarit Burden Interview (*p* < 0.001) and Motor Neuron Disease Behavioral Scale (*p* < 0.001) scores at baseline showed to be the best predictors of caregiver burden over time (27). Aside from the challenge that their behavioral and language impairment may present for caregivers, these patients require significant physical help with basic activities of daily living as the disease progresses (60). Caregivers are often ill-equipped to offer the type of care required to cope with the patient's motor and respiratory impairments and since it is physically demanding work, it is less likely that people within the caregiver's social network will help them with caregiving duties (61, 62). Worsening of symptoms and physical concerns may lead to increased stress, anxiety, and depression for caregivers, diminishing energy for leisure activities and time to fulfill their own needs (60). Caregivers who experience feelings of depression may find it even more challenging to cope with the caregiving demands placed on them and can neglect the patients (30, 62).

All types of FTD will follow a progressive fatal trajectory. Medical, financial, and end-of-life decisions need to be considered by caregivers, particularly in FTD-ALS where the disease progresses most rapidly (27). From diagnosis, all patients with FTD should receive information about advance care planning and caregiver assistance with understanding and consideration of the patient's wishes (63). Unfortunately, the training of specialists in palliative care is still insufficient, even in developed countries (64). Lack of planning can bring avoidable distress to caregivers since the ethical and emotional responsibilities to make such complex decisions are great (e.g., artificial nutrition/hydration, antibiotics, etc.). This is particularly true for caregivers with low education, greater financial burden, and limited access to providers and support services (65). In Latin America, these decisions are usually made through family consensus, sometimes even involving the extended family or respected members of the community (66). The role of religion is important in Latin American societies, and caregivers might seek a religious leader to also support their decisions (67). The absence of the support network formed by family and sometimes religious leaders when these decisions need to be made in emergency contexts can leave important emotional sequelae in the caregivers (68). It is important to note that only six countries in Latin America (Argentina, Brazil, Colombia, Mexico, Panama, and Uruguay) have specific legislation regarding an advance directive document and the requirements to create one, therefore, the family's discussion of these topics early in the course of these diseases is fundamental to avoid adverse outcomes in the caregiver (67).

### Next Steps in Supporting Caregiving for Patients With FTD in Latin America

Awareness of FTD in Latin America is scarce (7). Therefore, the first and most important point moving forward is to increase awareness of the disease at all levels, informing members of community-dwelling populations and healthcare providers alike. Generating awareness is fundamental to reducing stigma (69). Awareness should also be paired with the education of all sectors of the population to help caregivers to be informed about the disease (70). Table 1 highlights literature that included reportable outcomes following FTD caregiver interventions in different parts of the world. From this table we identified that caregivers of individuals with FTD benefitted from support groups, and education programs which addressed their specific needs with participants reporting improved knowledge and understanding of the disease, and valuing mutual support and sharing of coping strategies (73–77) The limitations for most of these interventions to be applied in Latin America is the lack of specialized providers, specialized resources and the inability to leverage technology that could include caregivers living in remote areas. On a large scale, two institutions, the Global Brain Health Institute^4^ as an international organization and the BrainLat Institute^5^ as a Latin American organization are taking some steps forward in training Latin American multidisciplinary professionals on FTD. The hope is to get a multi-directional effect in which they educate and raise awareness among other professionals, general population and policy makers and increase the creation of dementia resources in Latin America.

**Table 1:** Interventions for caregivers and implementation observations in Latin America.

**Type of FTD**	**Intervention**	**Author and country**	**Comments for implementation in Latin America**
FTD	Caregivers learned coping strategies such as problem-solving, reframing and seeking support in 16 weekly sessions or over 12 months, reducing caregiver burden.	AustraliaMioshi et al. (71)	Pros: - Low cost
			Cons: - Facilitators with long-term experience in FTD are required - Support systems need to exist to be considered an alternative
FTD	Caregivers attended 5 positive affect intervention sessions including themes of gratitude and mindfulness, resulting in reduced burden, depression, and perceived stress.	USADowling et al. (72)	Pros: - Intervention was tested online and in person - Short homework that keeps the participant engaged all week (Although this could unintentionally add burden to very collapsed caregivers)
			Cons: - Specialized nurses/psychologist are needed to deliver the intervention - High number of staff hours since it is an individual intervention
FTD	FTD caregivers attended 90-min support groups held on a weekly basis for 7 weeks, with improved caregiver coping and reduced social isolation.	GermanyDiehl et al. (73)	Pros: - Low costs and maximum number of opportunities to share experiences within caregivers
			Cons: - A specialized social worker and a physician were required in this intervention
bvFTD	Ten weekly 1-h FTD caregiver video-based support groups were held, with caregivers reporting greater emotional support and diminished burden.	CanadaMarziali and Climans (74)	Pros: - Hybrid program alternating facilitated and non-facilitated interventions sparing personnel time
			Cons: - Intervention tested in computer literate participants who had experience accessing the internet - A handbook and disease-specific support systems for referral are required
bvFTD	Caregivers attended a multimodal intervention over 6 months that included coping skills training and social support, reducing perceived stress and improved mood.	The NetherlandsGossink et al. (75)	Pros: - Improves sense of competence in caregivers
			Cons: - Trained psychologist and physician are required
PPA	Participants with PPA and their caregivers attended to 4 3-h PPA-specific group sessions that covered education, strategies for managing negative feelings and enhance successful communication, and opportunities for peer support. The intervention increased PPA knowledge, management of worry and low mood, reduced feelings of isolation, and increased feelings of support	AustraliaTaylor-Rubin et al. (76)	Pros: - Caregiver and patients assist together eliminating the need to look for a substitute caregiver - Adequate for all types of PPA
			Cons: - This intervention was conducted by a specialized and experienced speech pathologist
PPA	Five 90-min session where caregivers received an educational curriculum and peer support. An art component was added. An increase in PPA knowledge, self-confidence, coping abilities and sense of belonging were perceived	United KingdomMorhardt et al. (77)	Pros: - It gave enough space for caregivers to have a central role in the program - Caregivers were able to create part of the program according to their preferences.
			Cons: - It requires specialized profesionals to deliver the curriculum.

Specialized caregiver support groups and psychoeducational programs need to be subsidized to be available on a larger scale for caregivers and their funding should contemplate providing technological resources and support to people in remote areas in order to increase access. In the United States, the Association for Frontotemporal Degeneration^6^ formed by healthcare providers and caregivers represents a model that provides caregivers support resources and educational materials. In Spain, the Frontotemporal Dementia Association^7^ is a similar model that also provides resources to caregivers. These examples could be adapted to local needs in Latin America if the appropriate resources existed. The Alzheimer's Associations and the local groups (see text footnote 1–3) have taken the lead in supporting caregivers of patients with FTD on a smaller scale. These Associations must join efforts with caregivers, healthcare providers, and policymakers from all Latin America to advance FTD care in Latin America.

It is important to highlight the scarcity of literature on the caregiving for FTD patients in Latin America. Researchers' associations like the International Society for Frontotemporal Dementias^8^ and the Latin America and the Caribbean Consortium on Dementia^9^ keep raising awareness on this gap among their members and promote increasing research on this topic. Since FTD diagnosis is low in the region at this moment, even small group caregiver intervention strategies like the ones being put in place in different places in Latin America (see text footnote 1–3) are valuable, can inform the literature and provide guidelines to health providers in the region.

Health policies and infrastructure are fundamental to provide support to caregivers of persons with FTD. Even when national dementia plans from Costa Rica (78), Argentina (79), Uruguay (80), Chile (81), Perú, and Mexico (82) include caregiver support for dementia patients, in reality, more services and infrastructure are needed to fulfill the needs of caregivers, particularly in rural areas. The Uruguayan model of caregiver support functions as a first example to inform the region how policies on this topic can be made, implemented and improved over time to provide social, economic, and legal support to caregivers. It also provides a telehealth model that can benefit the sector of the population that has access to internet services and can be used as proof that such services need to be widely available for caregivers (40). Healthcare diplomacy is fundamental to create joint efforts that push policies forward to protect caregivers of patients with FTD in which all the aforementioned organization can act as stakeholders.

Finally, we must consider that despite the many barriers to care patients with FTD face in Latin America, there are also strengths inherent to its cultures and traditions. For instance, there is more emphasis on aging in the community and within the family, where it may be easier to find solidarity and tolerance even if the disease is not well-understood. Leveraging this as an advantage, it is very likely that in future circumstances, once better education and infrastructure exist, new models of care will emerge. Blending the resourcefulness that exists within the region while implementing broad changes that benefit patients with FTD and their caregivers. The goals are that patients and caregivers get the tools they need to seek care in the early stages of the disease, primary care providers become able to identify the disease and make an early referral, and specialists become able to provide a timely diagnosis that will help patients and caregivers obtain appropriate resources and plan for the future including palliative care. Ideally, services with adequately trained personnel become available and help reduce the burden of care for caregivers and the society becomes more educated and tolerant with patients with FTD reducing the stress of caregivers in social interaction situations.

## Data Availability Statement

The original contributions presented in the study are included in the article/supplementary material, further inquiries can be directed to the corresponding author.

## Author Contributions

SP-E conceived of the presented idea. All authors reviewed the existent literature, developed the idea, and contributed to the final manuscript.

## Conflict of Interest

The authors declare that the research was conducted in the absence of any commercial or financial relationships that could be construed as a potential conflict of interest.

## Footnotes

^1^https://ipqhc.org.br/

^2^https://www.ineco.org.ar/patologias/demencia-frontotemporal-dft/

^3^https://www.fundacionudea.com/sitio/neurociencias/

^4^www.ghi.og

^5^www.brainlat.uai.cl

^6^www.theaftd.org

^7^http://adef.es/site/

^8^www.isftd.org

^9^www.lac-cd.org/
